# A Warning to Doctors

**Published:** 1896-04

**Authors:** 


A Warning to Doctors.—We all know how difficult it is to keep back anything from one's wife. If she suspects that "hubby" knows anything of a confidential nature about any of his pretty patients she has a thousand ways of getting it out of him; and she always vows most earnestly that she will not tell. Too often hubby yields to the pressure. It may be that he compounds with his conscience, and justifies himself with the figure of speech "better half," or "man and wife are one;" and again, a secret may be so great as to to require two to keep it, he says. At any rate, has a doctor the right to take his wife into partnership with his professional secrets?

It seems that Dr. Wm. Playfair, the great English gynecologist, of London, was either of that way of thinking, or was weak enough to be wheedled by his wife into telling her a most important secret concerning a lady of the higher walks, and he paid dearly for his experience. The facts in the case are these: A Mr. Arthur Kitson was separated from his wife. Arthur's brother, Sir James Kitson, a millionaire, felt "sorry," we will be charitable enough to say, for the pretty grass widow, and was "making her an allowance," so the report runs. Dr. Playfair was her medical adviser and confident, and she confided to him something, which, from the sequel, must have been very important. This secret Mrs. Playfair got out of her husband, and woman like, had to get somebody to help *her* keep it, or it was too good to keep, and hence—"all over town," and Sir James withdraws the allowance. Result. Suit for damages, and verdict for widow; verdict $60,000!—and received by the audience with cheers; widow fainted. (Don't know whether the band played or not, suppose not.)

Who can imagine the first subsequent meeting between the doctor and his wife? Must have been interesting to both parties. Moral. Doctor, you and your wife may be "one," but when it comes to dividing professional secrets with your better half—at a cost of $60,000, to say nothing of peace and domestic felicity forever sacrificed—better hadn't do it.

